# MicroRNAs Related to TACE Treatment Response: A Review of the Literature from a Radiological Point of View

**DOI:** 10.3390/diagnostics12020374

**Published:** 2022-02-01

**Authors:** Alessandro Marco Bozzato, Paola Martingano, Roberta Antea Pozzi Mucelli, Marco Francesco Maria Cavallaro, Matteo Cesarotto, Cristina Marcello, Claudio Tiribelli, Devis Pascut, Riccardo Pizzolato, Fabio Pozzi Mucelli, Mauro Giuffrè, Lory Saveria Crocè, Maria Assunta Cova

**Affiliations:** 1 Department of Medical, Surgical and Health Sciences, University of Trieste, 34151 Trieste, Italy; matteocesarotto3@gmail.com (M.C.); cristina.marcello93@gmail.com (C.M.); mauro.giuffre@asugi.sanita.fvg.it (M.G.); lcroce@units.it (L.S.C.); m.cova@fmc.units.it (M.A.C.); 2 Department of Radiology, ASUGI, Ospedale di Cattinara, 34149 Trieste, Italy; pmartingano@sirm.org (P.M.); roberta.pozzimucelli@gmail.com (R.A.P.M.); riccardo.pizzolato@gmail.com (R.P.); fabio.pozzimucelli@asugi.sanita.fvg.it (F.P.M.); 3 Department of Radiology, ASUGI, Ospedale Maggiore, 34149 Trieste, Italy; mrc.cavallaro@virgilio.it; 4 Liver Research Center, Fondazione Italiana Fegato—ONLUS, Basovizza, 34149 Trieste, Italy; ctliver@fegato.it (C.T.); devis.pascut@fegato.it (D.P.)

**Keywords:** microRNA (miRNA), biomarkers, prognostic biomarkers, liver, liver cirrhosis, liver tumor, hepatocellular carcinoma (HCC), transarterial chemoembolization (TACE), treatment response criteria

## Abstract

Hepatocellular Carcinoma (HCC) is the sixth most common cancer in the world. Patients with intermediate stage (Barcelona Clinic Liver Cancer, B stage) hepatocellular carcinoma (HCC) have been able to benefit from TACE (transarterial chemoembolization) as a treatment option. MicroRNAs (miRNAs), i.e., a subclass of non-coding RNAs (ncRNAs), participate in post-transcriptional gene regulation processes and miRNA dysfunction has been associated with apoptosis resistance, cellular proliferation, tumor genesis, and progression. Only a few studies have investigated the role of miRNAs as biomarkers predicting TACE treatment response in HCC. Here, we review the studies’ characteristics from a radiological point of view, also correlating data with radiological images chosen from the cases of our institution.

## 1. Introduction

**Figure 1:**
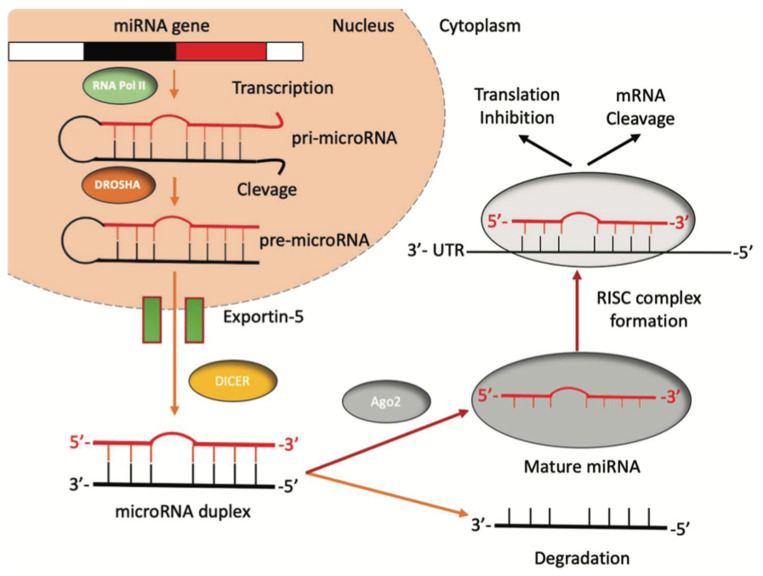
The biogenesis of miRNAs. miRNAs’ genes are transcribed by RNA polymerase II into a pri-miRNA containing one to six miRNA precursors. The double-strand RNA structure is recognized by a nuclear protein called Pasha (DGCR8), which binds the enzyme Drosha to release the pre-microRNA. Pre-miRNAs are exported from the nucleus to the cytoplasm using the shuttle of Exportin-5. Once in the cytoplasm, the pre-miRNA is cleaved by the RNase III Dicer, which interacts with the 3′-end and cuts the loop joining the 3′- and 5′-arms, producing a yet-not-final miRNA duplex (about 22 nucleotides in length). One strand of the mature miRNA is usually degraded, whereas one strand will become a mature miRNA bound to an RNA-mediated silencing complex (RISC). In this complex, the mature miRNA targets the 3′-UTR region of its target mRNA to regulate its translation.

Hepatocellular Carcinoma (HCC) is the sixth most-diagnosed cancer and the fourth cause of cancer-related death worldwide [1]. In 2018, a total of 841,000 new HCC had been diagnosed, with 782,000 new HCC-related deaths [1]. The highest incidence and mortality are registered in East Asia and Africa due to the geographical distribution of risk factors and etiological causes [2]. Over 90% of HCC cases arise in the setting of liver cirrhosis, with annual incidences of 1–6% [3]. Given that most cases of HCC occur in an identifiable population (i.e., those with chronic liver disease), many patients are diagnosed through surveillance tests (e.g., abdominal ultrasonography combined or not with serum α-fetoprotein levels) [3] that are not always efficient, thus leading to late diagnosis, especially in less developed countries. Once diagnosed, the treatment is assigned according to tumor stages, liver function abnormalities, and the expected benefits of major interventions, following the so-called Barcelona Clinic Liver Cancer (BCLC) staging system [4]. Transarterial chemoembolization (TACE), which remains as the recommended first-line therapy for patients with intermediate stage (BCLC B grade) HCC [5], has a poor long-term outcome [6]. Partial response is reported only in 15–55% of patients, with the reported five-year survival rates of TACE only ranging from 1–8% [7,8]. Thus, the presence of predictive algorithms might provide clinicians with a new tool for patient selection and prioritization. Therefore, it is urgent to explore new markers that can monitor disease progression or response to treatment. According to the most recent studies, microRNAs (miRNAs) have shown promising results in nucleic acid-based drugs, early diagnosis, and also disease monitoring [9]. MiRNAs are a subclass of non-coding RNAs approximately 22 nucleotides long, firstly discovered in Caenorhabditis elegans [10,11], that participate in post-transcriptional gene regulation processes by inhibiting the translation or favoring the degradation of the target messenger-RNA (mRNA) [12] (Figure 1). MiRNAs are found in most eukaryotes, including humans, and are present in all cell types and tissues. Even if miRNAs account for a small portion of the human genome (1–5%), they regulate around 30% of protein-coding genes. It has been known that miRNA dysfunction is associated with apoptosis resistance and uncontrolled cellular proliferation. Emerging evidence also suggests that miRNAs dysregulation may be central in tumor genesis and progression [13].

Only a few studies [14,15,16,17,18,19,20,21,22,23,24,25,26,27,28,29,30] have reported evidence on the role of miRNAs as TACE treatment response biomarkers in HCC. Therefore, the aim of the current paper is to review these studies from a radiological point of view, also showing explanatory images from our series.

## 2. TACE Technique

In the last three decades, patients with BCLC-B stage HCC [31,32] have been able to benefit from TACE as a treatment option. This treatment has been used as a neoadjuvant therapy to bridge patients with early-stage HCC to liver transplantation or downstage HCC to resectability [33,34]. TACE aims to induce tumor necrosis thanks to a cytotoxic effect and tissue ischemia, which is possible since HCC has a greater arterial vascularization compared to the surrounding parenchyma. There are mainly two TACE techniques, namely conventional TACE (cTACE), which consists of intra-arterial administration of an embolic agent and a cytotoxic drug (i.e., doxorubicin, epirubicin, and cisplatin), emulsified with an iodized oil such as Lipiodol emulsion (Lipiodol^®^ Ultra-Fluid, Guerbet) [35], and TACE, which is done with synthetic microspheres preloaded with a chemotherapy drug (drug-eluting beads, DEBs) [36]. The most common adverse reaction for both DEB-TACE and c-TACE is the post-embolization syndrome which consists of pain, fever, nausea, and vomiting. RCTs did not demonstrate any significant difference between the two techniques in terms of safety endpoints [37,38].

**Figure 2:**
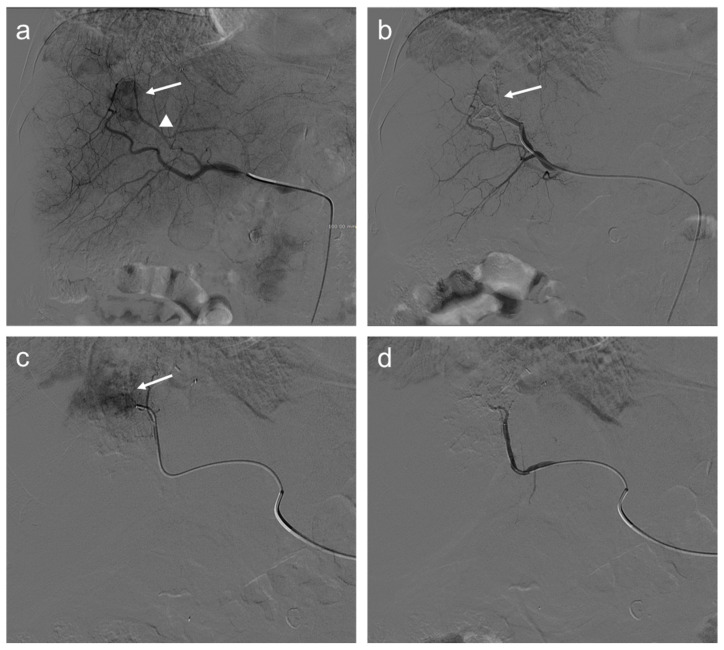
Seventy-two-year-old man with HCC undergoes TACE procedure: selective angiography (**a**) demonstrates a hypervascular mass in segment VIII (arrow) supplied by a hepatic artery branch (arrowhead); (**b**) after transarterial chemoembolization (TACE), a reduction of the size of the lesion is appreciated (arrow); selective angiography of the branch of the left hepatic artery (**c**) supplying the same lesion shows persistent enhancing tumor (arrow); and angiography after another injection of doxorubicin and synthetic microspheres (**d**) where the lesion is no longer visible.

Nevertheless, several studies demonstrated that an increase in locoregional hepatic toxicity was associated with DEB-TACE compared to cTACE. Global hepatic damages, intrahepatic biloma, and biliary injuries were more often described after DEB-TACE in patients with a higher prothrombin time (PT) value, suggesting that cTACE might be more suitable in patients with localized tumors and less advanced cirrhosis. Meanwhile, in patients with advanced age, poor liver function, or large and/or bilobar tumors, DEB-TACE appears to be more favorable. However, this added toxicity of DEB-TACE did not translate into a reduction of treatment efficacy, as both techniques demonstrated similar tumor responses [39]. Indeed, in the last years, several randomized controlled trials (RCTs) were conducted in Europe, showing no significant differences between cTACE and DEB-TACE in terms of local tumor control and/or survival [40,41,42]. Among these trials, the Precision Italia Study Group ended because no significant differences were observed in tumor response and the median time-to-progression was nine months in both arms [42]. Nevertheless, supplementary analyses of Precision V multicenter RCT demonstrated that in 67% of patients with more advanced disease, the incidences of an objective response and disease control were significantly higher in the DEB-TACE than c-TACE group (*p* = 0.038 and *p* = 0.026, respectively) [40]. Figure 2 shows an example of the TACE procedure, as performed in our institution.

## 3. Treatment Response Criteria

**Figure 3:**
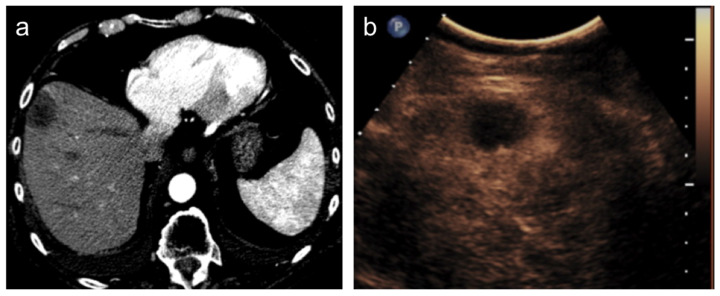
Hepatocellular carcinoma treated with transarterial chemoembolization (TACE) with complete response. After TACE, no residual enhancement is seen in the arterial phase on contrast-enhanced CT scan (**a**) and ultrasound (**b**).

After transcatheter HCC therapies, such as TACE, the assessment of tumor response is achieved by imaging since the persistence of the viable tumor is recognizable as an enhancing tissue in or along with the treated lesion. Contrast-enhanced ultrasound (CEUS) has shown good diagnostic performance in demonstrating the persistence of viable tissue [43] but it is suitable only for the evaluation of a single lesion and it suffers from all known US limitations, such as patient-body habitus, thus its use is limited to selected patients [44]. Furthermore, it is not performed in all institutions. Contrast-enhanced multiphasic CT or MR is the method of choice in post-treatment evaluations and they are usually performed after one month from the procedure and then every 3 to 6 months [45]. In the case of complete response, the lesion will show no enhancement in all phases (Figure 3).

**Figure 4:**
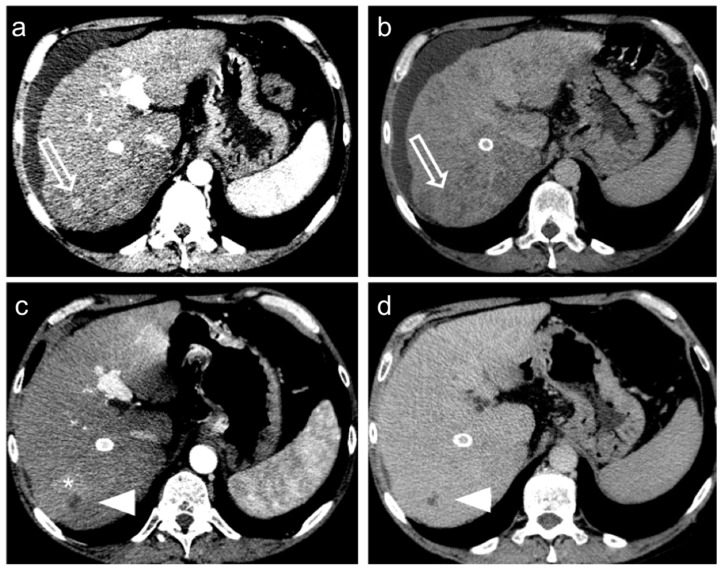
Hepatocellular carcinoma treated with transarterial chemoembolization (TACE) with a complete response: pre-treatment contrast-enhanced CT (**a**,**b**) shows a 12 mm nodule (arrow) in segment 7 with arterial enhancement (**a**) and late wash-out (**b**). After TACE (**c**,**d**), no residual enhancement is seen in the arterial phase (arrowhead in (**c**)) and there is no wash-out in the late phase (arrowhead in (**d**)). A geographic region of altered enhancement surrounding the treated lesion in the arterial phase (*) is considered normal.

A thin regular rim of enhancement at the periphery of the treated lesion is considered normal (Figure 4) but in these cases, the use of coronal and sagittal planes increases the detection of the small foci of tumor persistence at the borders.

**Figure 5:**
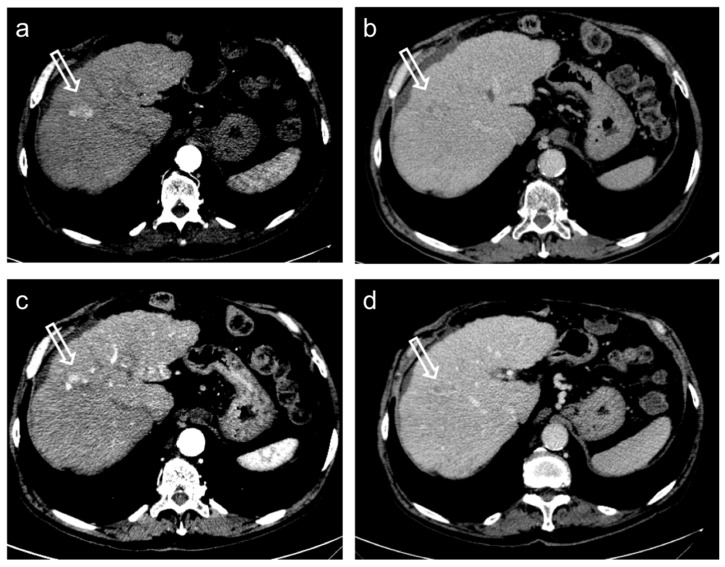
Hepatocellular carcinoma treated with transarterial chemoembolization (TACE) with partial response. Pre-treatment contrast-enhanced CT (**a**,**b**) shows a 24 mm nodule (arrow) in segment 8 with arterial hyper-enhancement (**a**) and late wash-out (**b**). After TACE (**c**,**d**), the presence of nodular hyper-enhancement in the arterial phase (arrow in (**c**)) with wash-out in the late phase (arrow in (**d**)) demonstrate the residual viable tumor consistent with a partial response to treatment.

Different criteria have been developed to evaluate tumor response based on post-treatment enhancement [45]. The widely used Response Evaluation Criteria in Solid Tumours (RECIST 1.1.) [46] is based only on size criteria and thus they failed to represent real oncological benefits for therapies focalized to reduce tumor vascularity rather than obtain mass shrinkage. The Modified Response Evaluation Criteria in Solid Tumours (mRECIST) [47] better represent locoregional treatment response, focusing on viable tumor presence. A tumoral tissue residual is recognized by its uptake in the arterial phase of contrast-enhanced images while the non-enhancing part of the lesion is considered responding to treatment also if stable in size (Figure 5).

This system is focalized on a per-patient evaluation, thus it is possible to select a maximum of two liver target lesions, evaluating tumor response. Partial response (PR) corresponds to a ≥30% decrease in the sum of the diameters of the viable tissue in target lesions while a progressive disease (PD) is considered as an increase equal to or greater than 20% of the sum of viable tissue compared to pre-treatment imaging. Complete response (CR) and stable disease (SD) correspond to “disappearance of any intratumoral arterial enhancement in all target lesions” and “any cases that do not qualify for either partial response or progressive disease”, respectively. A per-lesion evaluation is instead the approach of the Liver Imaging Reporting and Data System (LI-RADS) algorithm [48]. It focuses on the response of each treated lesion separately and response is categorized as complete (not viable residual tumor) or not complete (presence of any size of viable tissue). To increase sensitivity, the viable residual tumor could be recognized not only by the presence of a nodular arterial enhancement but also by the presence of a nodular wash-out in the portal venous or equilibrium phase, or when an altered enhancement, similar to that of the lesion before the treatment, is still recognizable.

## 4. MiRNA and TACE

Given the extreme variability of TACE treatment response, it is urgent to find pre-treatment biomarkers that allow for identifying a priori patient-responders from non-responders for a better selection of the patients in clinical practice.

Few studies [14,15,16,17,18,19,20,21,22,23,24,25,26,27,28,29,30] have investigated the role of miRNAs as TACE treatment response biomarkers in HCC and have demonstrated that several miRNAs (miR-1271, miR-4492, miR-214, miR-125b, miR-26a, miR-106b, miR-107, miR-133b, miR-590-5p, miR-21, miR-29a-3p, miR-122, miR-1268a, miR-199a/b-3p, miR-196a2 rs11614913, miR-499a rs3746444, miR-335, miR-10a, miR-23a, miR-24, miR-27a, miR-30c, miR-30e, miR-31, miR-200 a/b, miR-1285-3p, miR-4741, and miR-210) were associated with TACE treatment response. The studies are extremely heterogeneous and difficult to compare with each other in terms of aims and outcomes. Moreover, from a radiological point of view, TACE techniques and treatment response criteria are remarkably different from study to study. Therefore, to make reading easier, the studies’ characteristics, including the number of patients, sample source, method, TACE technique, treatment response criteria, primary outcomes, and the miRNAs related to TACE treatment response, are summarized in Table 1.

Even though these studies appear promising, to confirm their reproducibility, multicenter validation trials have to be conducted in a larger cohort considering that the miRNA profile can be influenced by individual variabilities, such as gender, race, and tumor etiology. In addition, the comparison of different studies sometimes can be difficult because a homogenous methodological protocol to process and analyze samples has not yet been reached. Further evidence must be provided before miRNA can be routinely used in clinical practice.

**Figure 6:**
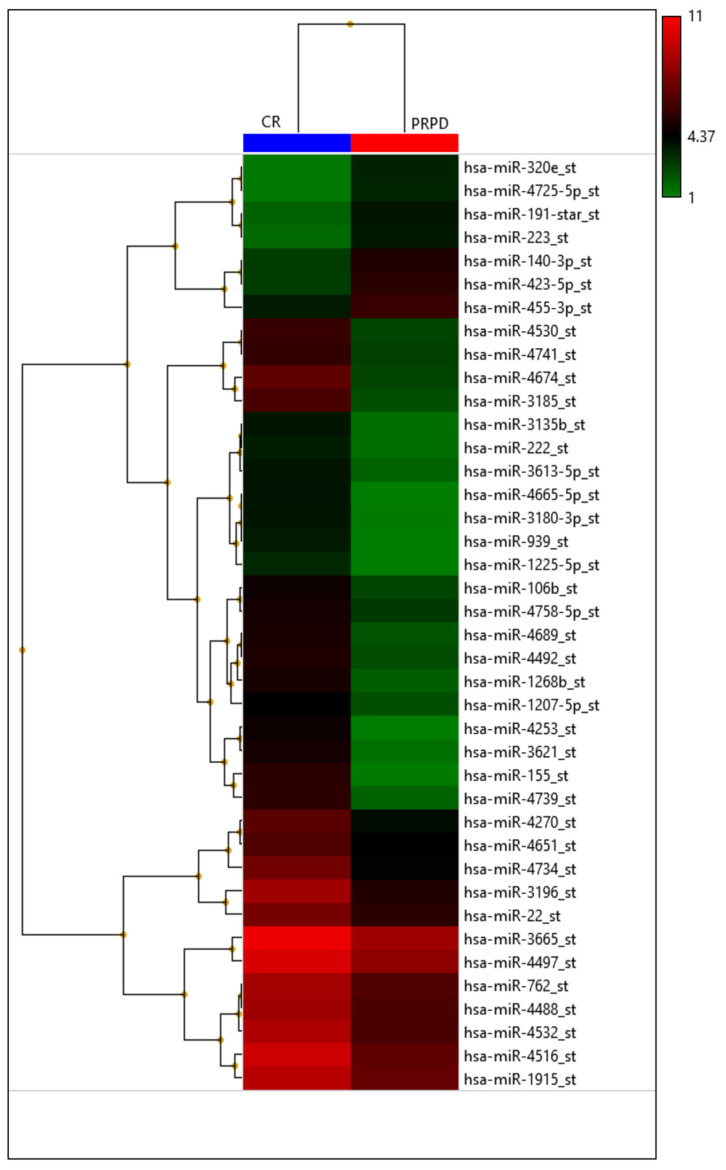
Heatmap with the pseudo-color scale underneath the differentially expressed miRNAs. Log2-transformed microarray signal is considered in the comparison between complete responder (CR; blue bar), partial responder, and progressive diseases (PRPD; red bar) patients. Unsupervised hierarchical clustering was used to order miRNAs. The sample tree with optimized leaf-ordering was drawn using Euclidean distances and average linkages for cluster-to-cluster distance. CR = complete responder and PRPD = partial responder and progressive diseases.

**Figure 7:**
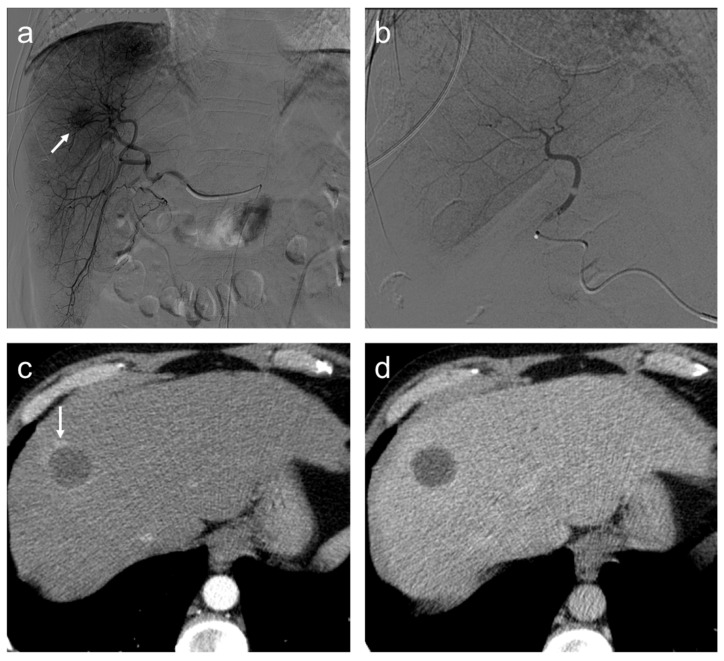
Complete response. Seventy-seven-year-old man, affected with HCC, expresses high miR-4492 levels at the time of diagnosis and undergoes TACE procedure: (**a**) selective angiography shows a hypervascular mass in segment VIII (arrow); (**b**) after TACE, the lesion was no longer visible; and (**c**) after the procedure, (**c**,**d**), no residual enhancement is seen in the arterial phase and there is no wash-out in the late phase. A geographic region of altered enhancement surrounding the treated lesion in the arterial phase (arrow) is considered normal.

**Figure 8:**
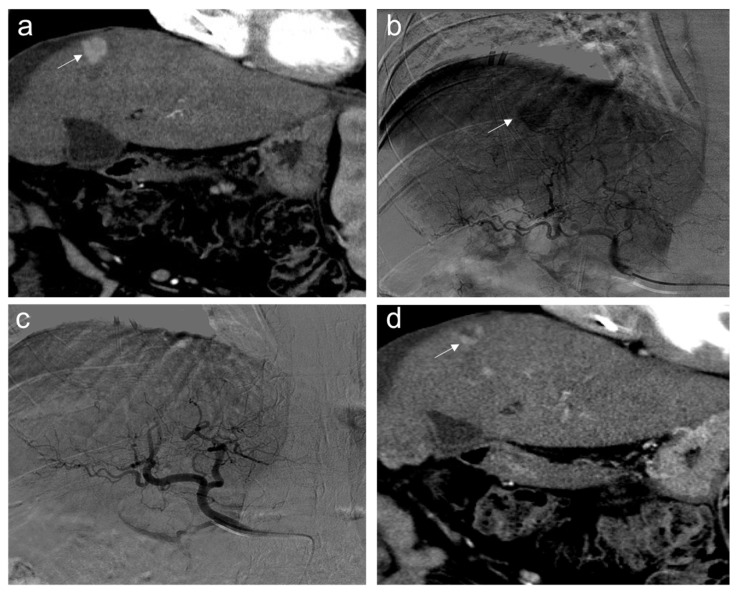
Partial response. Sixty-five-year-old man, affected with HCC, expresses low miR-4492 levels at the time of diagnosis and undergoes TACE procedure: (**a**) pre-treatment arterial contrast-enhanced CT shows a 22 mm nodule (arrow) in segment 8 with arterial hyper-enhancement; (**b**) selective angiography confirms a hypervascular mass in segment VIII (arrow); (**c**) after TACE, the lesion was no longer visible; and (**d**) one month after the procedure, the presence of nodular hyper-enhancement in the arterial phase (arrow) demonstrates the residual viable tumor consistent with a partial response to treatment.

In our institution, 46 patients treated with TACE were grouped according to therapy response in complete responders (CR) and partial responders or progressive disease (PRPD) following mRECIST criteria. It has been demonstrated that serum miR-4492 levels were associated with TACE response (Figure 6); indeed, miR-4492 levels at the time of diagnosis of HCC were 2.67-folds higher in patients with complete response to TACE (Figure 7) compared to patients with partial response or progressive disease (Figure 8). Therefore, the utilization of miR-4492 as a circulating biomarker might support the idea of individualized treatment strategies based on the risk prediction model to predict TACE outcomes [15].

## 5. Conclusions and Future Perspectives

TACE has a poor long-term outcome with partial response reported only in 15–55% of patients and five-year survival rates of TACE ranging from 1–8%. Therefore, the careful selection of patients undergoing TACE treatment would allow got lower complication rates and lower risk of failure.

Although it is necessary to prospectively validate these studies in large-scale trials to confirm these findings before clinical implementation, the use of miRNAs by radiologists and clinicians as biomarkers may be a valuable tool for predicting response to TACE, supporting the idea of individualized treatment strategies based on the risk prediction model.

## Figures and Tables

**Table 1:** Studies’ characteristics, including the authors (years), number of patients, miRNA related to TACE treatment response, sample source, method, TACE technique, treatment response criteria, and main endpoint.

Authors (Year)	Number of Patients	miRNA	Sample Source	Method	TACE Technique	Treatment Response Criteria	Main Endpoint
Guo Z. et al. (2020) [14]	162	miR-1271	serum	qRT-PCR	ethiodized oil, fluorourea glycoside, oxaliplatin, and polyvinyl alcohol	mRECIST	The level of miR-1271 in remission patients was significantly increased after TACE
Pratama M.Y. et al. (2020) [15]	46	miR-4492	serum	qRT-PCR	NM	mRECIST	Serum miR-4492 levels were associated with TACE response; indeed, miR-4492 levels at the time of diagnosis of HCC were 2.67-folds higher in patients with complete response to TACE compared to patients with partial response or progressive disease
Tang L. et al. (2020) [16]	87	miR-214	serum	qRT-PCR	chemotherapy drugs and super-liquid iodized oil-mixed emulsion	RECIST	High serum miR-214 levels one day before TACE were associated with disease control (complete remission, partial remission, and stability) one month after TACE, whereas low serum miR-214 levels one day before the procedure were related to disease progression one month after TACE. Nevertheless, one month after the treatment, the serum miR-214 levels were increased in both groups (patients with disease control and patients with disease progression) compared with before treatment
Wei X. et al. (2020) [17]	61	miR-125b	tissue samples	qRT-PCR	chemotherapeutic agents (including doxorubicin, epidoxorubicin, cisplatin, and mitomycin C) and ethiodized oil (5–10 mL)	mRECIST	High miR-125b levels were associated with survival benefit from adjuvant TACE
Ali H.E. et al. (2019) [18]	51	miR-26a, 106b, 107, and 133b	serum	qRT-PCR	doxorubicin (100 mg) and ethiodized oil	RECIST 1.1	High serum miRNA-106b, miRNA-107, and miRNA-133b levels were associated with TACE response, while high serum miRNA-26a levels were associated with no response to TACE
Chen M. et al. (2018) [19]	20	miR-590-5p	tissue samples	qRT-PCR	NM	NM	High miR-590-5p expression was associated with a favorable chemotherapy response
Kim S.S. et al. (2018) [20]	198	combination of miR-21, 26a, and 29a-3p	plasma	qRT-PCR	doxorubicin (50 mg) and ethiodized oil (10 mL), followed by embolization with gelatin sponge particles	mRECIST	Individual or combined plasma miR-21, miR-26a, and miR-29a-3p expression levels before the treatment were not significantly associated with liver transplantation-free survival or overall TACE refractoriness. Nevertheless, the combination of high miR21, high miR-26a, and low miR-29a-3p plasma levels were independent predictive factors for early TACE refractoriness, defined as TACE refractoriness within 1 year from the first TACE treatment
Suehiro T. et al. (2018) [21]	75	miR-122	serum	qRT-PCR	epirubicin hydrochloride or miriplatin hydrate, mitomycin C, ethiodized oil, and contrast agent, followed by embolization with gelatin sponge particles	NM	Low miR-122 ratio (serum miR expression after TACE/serum miR expression before TACE) was associated with significantly lower disease-specific survival
Kim S.S. et al. (2017) [22]	177	miR-122	plasma	qRT-PCR	doxorubicin (50 mg) and ethiodized oil (10 mL), followed by embolization with gelatin sponge particles	mRECIST	High plasma miR-122 expression before TACE was correlated with early TACE refractoriness, defined as TACE refractoriness within 1 year from the first TACE treatment
Lu Y.L. et al. (2016) [23]	411	miR-1268a	tissue samples	qRT-PCR	doxorubicin, cisplatin, and ethiodized oil, followed by embolization with gelatin foam or polyvinyl alcohol	NM	Low miRNA-1268a expression four weeks before the procedure predicted increased overall survival and relapse-free survival
Luo Z. et al. (2016) [24]	132	miR-199a/b-3p	serum	qRT-PCR	adriamycin (20–50 mg), ethiodized oil (5–20 mL), and contrast agent, followed by embolization with polyvinyl alcohol particles	mRECIST	High miR-199a/b-3p expression levels before TACE were related with CR and PR. Moreover, the decrease of miR-199a/b-3p levels 3–5 days and four weeks after the procedure were higher in patients with CR and PR than in the other ones
Qiu G.P. et al. (2016) [25]	507	CC genotype and C allele in miR-196a2 rs11614913, and GG genotype and G allele in miR-499a rs3746444	whole-blood	qRT-PCR	Epirubicin (40–80 mg) and hydroxycamptothecin (20–30 mg). Ethiodized oil (5–20 mL), and gelatin sponge particles were also injected in patients with less than six points of the Child-Pugh score	RECIST 1.1	The frequency of CC genotype and C allele in miR-196a2 rs11614913, as well as the GG genotype and G allele in miR-499a rs3746444 was higher in patients with stable disease and progression disease after TACE
Cui L. et al. (2015) [26]	125	miR-335	serum	qRT-PCR	carboplatin (300 mg), epirubicin (50 mg), mitomycin C (8 mg), and ethiodized oil (5 mL), followed by embolization with gelatin sponge particles	RECIST	The serum miR-335 expression thirty days after TACE may help in distinguishing good responders (CR and PR) from poor responders (SD and PD) with AUC of 0,922, specificity of 87%, and sensitivity of 77%. Indeed, low serum miR-335 levels thirty days after the procedure were correlated with poor response as well as decreased overall survival and time to progression in these patients
El-Halawany M.S. et al. (2015) [27]	15	miR-10a, 23a, 24, 26a, 27a, 30c, 30e, 31, 106b, 133-b, 199a-3p, and miR-200 b	tissue samples	qRT-PCR	cisplatin (50 mg), doxorubicin (50 mg), and ethiodized oil	RECIST	Pretreatment expression of this panel of 12 miRNAs was higher in non-responders to TACE
Liu J. et al. (2015) [28]	97	miR-1285-3p and miR-4741	plasma	qRT-PCR	epirubicin (30 mg), cyano-camptothecin (30 mg), and cisplatin (40 mg)	NM	Low miR-1285-3p and miR-4741 levels in HCC patients before the TACE procedure were correlated with poor response to the treatment
Liu M. et al. (2014) [29]	136	miR-200a	serum	qRT-PCR	adriamycin (20–50 mg), ethiodized oil (5–20 mL), and contrast medium, followed by embolization with gelatin sponge particles	NM	The potential role of miR-200a as an independent prognostic factor associated with disease outcome in HCC patients treated with TACE was described. Indeed, high serum miR-200a levels in patients with HCC before TACE treatment were associated with decreased survival
Zhan M. et al. (2014) [30]	113	miR-210	serum	qRT-PCR	Oxaliplatin (135 mg) and epirubicin (30–40 mg). In case of incomplete embolization, gelatin sponge particles were also injected	mRECIST	High miR-210 serum levels before TACE were associated with SD and PD, and the increase of miR-210 serum levels four weeks after the treatment were higher in patients with SD and PD

qRT-PCR (quantitative Reverse Transcription-Polymerase Chain Reaction), mRECIST (Response Evaluation Criteria in Solid Tumours), RECIST (Response Evaluation Criteria in Solid Tumours), TACE (transarterial chemoembolization), NM (not mentioned), CR (complete response), PR (partial response), SD (stable disease), and PD (progressive disease).

## Data Availability

Not applicable.
